# Expression of calcium-binding proteins and selected neuropeptides in the human, chimpanzee, and crab-eating macaque claustrum

**DOI:** 10.3389/fnsys.2014.00099

**Published:** 2014-05-26

**Authors:** Andrea Pirone, Maura Castagna, Alberto Granato, Antonella Peruffo, Francesca Quilici, Laura Cavicchioli, Ilaria Piano, Carla Lenzi, Bruno Cozzi

**Affiliations:** ^1^Department of Veterinary Sciences, University of PisaPisa, Italy; ^2^Department of Translational Resource on New Technologies in Medicine and Surgery, University of PisaPisa, Italy; ^3^Department of Psychology, Catholic UniversityMilan, Italy; ^4^Department of Comparative Biomedicine and Food Science, University of PadovaPadova, Italy; ^5^Department of Pharmacy, University of PisaPisa, Italy

**Keywords:** claustrum, calretinin, parvalbumin, somatostatin, NPY, human, chimpanzee, monkey

## Abstract

The claustrum is present in all mammalian species examined so far and its morphology, chemoarchitecture, physiology, phylogenesis and ontogenesis are still a matter of debate. Several morphologically distinct types of immunostained cells were described in different mammalian species. To date, a comparative study on the neurochemical organization of the human and non-human primates claustrum has not been fully described yet, partially due to technical reasons linked to the postmortem sampling interval. The present study analyze the localization and morphology of neurons expressing parvalbumin (PV), calretinin (CR), NPY, and somatostatin (SOM) in the claustrum of man (# 5), chimpanzee (# 1) and crab-eating monkey (# 3). Immunoreactivity for the used markers was observed in neuronal cell bodies and processes distributed throughout the anterior-posterior extent of human, chimpanzee and macaque claustrum. Both CR- and PV-immunoreactive (ir) neurons were mostly localized in the central and ventral region of the claustrum of the three species while SOM- and NPY-ir neurons seemed to be equally distributed throughout the ventral-dorsal extent. In the chimpanzee claustrum SOM-ir elements were not observed. No co-localization of PV with CR was found, thus suggesting the existence of two non-overlapping populations of PV and CR-ir interneurons. The expression of most proteins (CR, PV, NPY), was similar in all species. The only exception was the absence of SOM-ir elements in the claustrum of the chimpanzee, likely due to species specific variability. Our data suggest a possible common structural organization shared with the adjacent insular region, a further element that emphasizes a possible common ontogeny of the claustrum and the neocortex.

## Introduction

The word claustrum comes from the Latin term that indicates a hidden place, here referred to the position of the structure that is placed between the inner surface of the insular cortex and the outer surface of the putamen. The claustrum is a symmetrical, thin, and irregular sheet of gray matter present in all mammalian species examined so far, including man (Kowianski et al., 1999). Its phylogenesis and ontogenesis are still a matter of debate (Edelstein and Denaro, 2004; Pirone et al., 2012). Projections to the primary somatosensory cortex have been demonstrated in the monkey claustrum (Minciacchi et al., 1991) and reciprocal diffuse connections with the cortex have been shown in several species (Carman et al., 1964; LeVay and Sherk, 1981; Carey and Neal, 1985; Dinopoulos et al., 1992). However the existence of functional subunits of the claustrum, based on topography of cortical projections or segregation of neural cell types, remains uncertain.

Calbindin-28 KD (CB), parvalbumin (PV), and calretinin (CR) belong to the EF-hand family of the calcium binding proteins (CBPs; Baimbridge et al., 1992). The CBPs are involved in many cellular physiological processes mediated by Ca^2+^ (Miller, 1991) and these neural markers are widely expressed also in the brain of vertebrates (Jande et al., 1981; Parmentier et al., 1987; Baimbridge et al., 1992; Andressen et al., 1993; Crespo et al., 1999; Dávila et al., 2000; Díaz-Regueira and Anadón, 2000; Milán and Puelles, 2000; Castro et al., 2003; Morona and González, 2008). The immunohistochemical distribution of the CBPs is an excellent tool to highlight the relationship between function and structure most notably in the thalamus (Jones and Hendry, 1989; Rausell and Jones, 1991; Rausell et al., 1992; Cusick et al., 1993) and in the brainstem (Parvizi and Damasio, 2003). A series of recent studies focused on the expression of CBPs in the claustrum of different species (Kowianski et al., 1999; Real et al., 2003; Wojcik et al., 2004; Rahman and Baizer, 2007). In particular, in the monkey claustrum, immunoreactivity to the CBPs revealed no functional segregation or structural heterogeneity (Reynhout and Baizer, 1999). Reactivity to PV has been reported in a recent detailed study in the human claustrum (Hinova-Palova et al., 2013).

CBPs and neuropeptides have been used as markers that distinguish among types of cortical interneurons (DeFelipe et al., 1989; Hendry et al., 1989; Rogers, 1989; DeFelipe, 1993, 1997; Cauli et al., 1997; Gonchar and Burkhalter, 1997; Somogyi and Klausberger, 2005; Ascoli et al., 2008). Moreover, it has been demonstrated that inhibitory neurons expressing CBPs contain also neuropeptides such as: somatostatin (SOM), vasoactive intestinal peptide (VIP), cholecystokinin (CCK), and neuropeptide Y (NPY; Xu et al., 2006).

The topography of immunoreactive interneurons is relevant for a better understanding of the chemoarchitecture of the claustrum and its functional role. Little is known about the function of the claustrum and specific systematic studies are sparse. For these reasons, the aim of the present study is to characterize the distribution of CBPs in neural cells of the human, chimpanzee and crab-eating monkey claustrum, and compare them with the distribution of NPY and SOM in the same species.

## Materials and methods

### Tissue samples

In this study we used archival samples obtained from five patients of different sex and age, with no history of psychiatric or neurological disorder. The average age was 59.6 years and the average post-mortem delay was 26 h. The samples consisted of blocks approx. 5 cm thick, including the claustrum, surrounded by portions of the adjoining structures (extreme and external capsules, insular cortex, putamen). The samples were carefully dissected during post-mortem procedures performed by qualified pathologists at the S. Chiara Hospital, University of Pisa. The brain samples were removed within routine diagnostic scopes, following a procedure approved by the Ethic Committee of the University of Pisa (protocol number 3482). The blocks were fixed by immersion in buffered formalin, washed in phosphate saline buffer (PBS) 0.1 M, pH 7.4 and processed for paraffin embedding. Reference sections were stained either with Nissl or Luxol Fast Blue stains.

We also considered samples of primate brains. To this effect we examined the claustrum of an adult male chimpanzee (*Pan troglodytes*, Blumenbach, 1775) living in a zoological park, whose body was forwarded to the Department of Comparative Biomedicine and Food Science of the University of Padova for post-mortem diagnosis. The time interval between death and removal of the brain cannot be determined precisely, as the animal was found dead by the wardens on the morning round. The cause of death was not neurological. Samples from the chimpanzee brain were treated with the same protocol used for the human samples. The monkey claustrum was sampled from the brains of the three crab-eating macaques (*Macaca fascicularis*, Raffles, 1821) stored in the archives of the same Department. The brains were initially perfused with buffered formalin and removed from animals formerly employed for a translational transplant research authorized by the University Ethical Committee. Use of archival samples is encouraged based on the EU Directive 2010/63/ of 22 September 2010 on the protection of animals used for scientific purposes (Introduction section).^1^ After repeated washings in PBS, perfusion-fixed samples from the crab-eating monkey brains were processed for paraffin embedding. Archival brain samples of the rat cortex stored at the Department of Veterinary Sciences of the University of Pisa were used as a positive control for CBPs immunostaining (see below).

### Immunohistochemistry

A rabbit polyclonal anti- CR antibody (H-45: sc-50453; Santa Cruz Biotech., Inc., Santa Cruz, CA; dilution 1:200), a mouse monoclonal anti-PV antibody (Clone PA-235, Cat. # P-3171, Sigma-Aldrich, St. Louis, MO, USA; dilution 1:3000), a mouse monoclonal anti-CB-D-28K antibody (Clone CB-955, Cat. # C9848, Sigma-Aldrich, St. Louis, MO, USA; dilution 1:3000) a rabbit polyclonal anti NPY antibody (ab30914, Abcam; dilution 1:3000), and a rabbit polyclonal anti-SOM antibody (ab103790, Abcam; dilution 1:700) were used in this study. Epitope retrieval was carried out at 120°C in a pressure cooker for 5 min using a Tris/EDTA buffer pH 9.0. Sections were rinsed in PBS and incubated in 1% H_2_O_2_-PBS for 10 min, then pre-incubated in PBS with 0.3% Triton X-100 (TX) (Sigma-Aldrich, St. Louis, MO, USA) and 5% normal goat serum (Vector Labs, Burlingame, CA) to reduce non-specific staining. Next, sections were incubated overnight in a humid chamber at 4°C with the primary antibody diluted in PBS with 0.3% TX and 1% normal goat serum. After several washings in PBS, sections were incubated for 1 h at room temperature in biotinylated goat anti-rabbit immunoglobulins (for CR, NPY and SOM) and biotinylated goat anti-mouse immunoglobulins (for PV and CB) (Vector Labs, Burlingame, CA), diluted 1:300 in PBS. Sections were then washed for 3 × 10 min in PBS, and incubated for 1 h at room temperature in avidin-biotin-horseradish peroxidase complex (ABC; Vector Labs, Burlingame, CA), diluted 1:125 in PBS. After washing for 3 × 10 min in Tris/HCl (pH 7.6), peroxidase activity was detected by incubation in a solution of 0.125 mg/ml diaminobenzidine (Sigma-Aldrich, St. Louis, MO, USA) and 0.1% H_2_O_2_ in the same buffer for 10 min.

### CBPs protein sequences in the three species

The amino-acid composition of CR is highly preserved in mammals. The antibody that we used recognizes human epitope aa 123–167, with a minimal correspondence score of 99.43% considering clusta1W2,^2^ or >98%^3^ if considering *Macaca mulatta* instead of *Macaca fascicularis*. The PV amino-acid sequence is also maintained with a minimum of 97.27% correspondence between the species that we examined.^3^ The antibody that we used was a monoclonal raised against the segments of the protein sequence conserved in man and macaque and was extensively validated in man, monkey and rodents (for reference see Saleem et al., 2007). Conservation of the CB amino-acid sequence between the three primates is >98.85^3^ and >98.47% with the sequence of the bovine kidney used to produce the antibody.

The specificity of the immunohistochemical staining was field tested in repeated trials as follows: substitution of either the antibody, the anti-rabbit IgG, or the ABC complex by PBS or non-immune serum. Under these conditions staining was abolished.

Moreover, cryostat sections (15–20 µm) of a rat brain perfused with 4% paraformaldehyde were employed as positive control.

### Procedure for PV and CR-ir neurons count

Three evenly spaced 3 µm thick coronal sections were cut through the claustral region (and including also parts of the insular cortex) of each brain and mounted on positively charged slides. In each section the whole area of the claustrum was analyzed at 10x. In all the microscope fields captured with 10x objective the number of positive cells was counted employing also a 25x objective to better detect positive neurons. To compare immunoreactive neuronal subpopulations containing PV and CR, the neuronal density as the number of cells per square millimeter was estimated. Additional coronal sections were also stained with Cresyl violet and Luxol Fast Blue for comparison. All photos were taken with a light microscope (Leitz Diaplan, Wetzlar, Germany) connected to a PC via a Nikon digital system (Digital Sight DS-U1).

### Immunofluorescence co-localization

Two sections for each species (one rostral and one caudal) were washed 3 × 10 min in PBS, permeabilized and blocked with PBS + 1% bovine serum albumin (BSA) + 0.3% Triton X-100 in a humid chamber at room temperature for 45 min. Sections were then incubated overnight in a humid chamber at 4°C using a combination of rabbit polyclonal anti-CR antibody and a mouse monoclonal anti-PV antibody (1:200/1:2000), diluted in PBS + 1% BSA + 0.03% TritonX-100 (PBS-BT). After washing for 3 × 10 min in PBS, the slides were incubated in a combination of secondary antibodies: anti-mouse Alexa 488 (1:500) and anti-rabbit Rhodamin Red-x (1:1000) (Invitrogen, Carlsbad, CA, USA). The sections were washed for 3 × 10 min in PBS and mounted in Vectashield (Vector Labs). Slides were examined with a Leica TCS-NT confocal microscope equipped with a krypton–argon laser.

The specificity of the immunohistochemical staining was tested in repeated trials by replacing either the primary or the secondary antibody with PBS.

## Results

**Figure 1:**
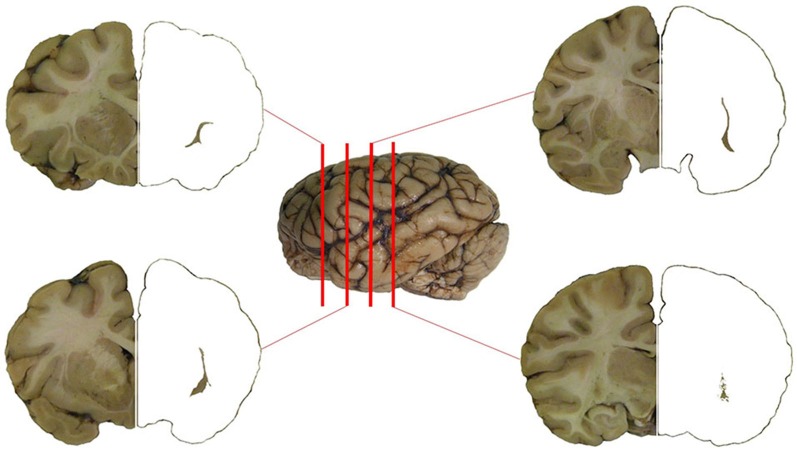
**The claustrum of the chimpanzee in four subsequent sections of the brain**. Position of the section is represented against the left side of the brain. The claustrum has been isolated on the right side of the four sections.

The planes of section represented in Figure 1 were regularly applied to the analysis of the chemical neuroanatomy of the claustrum in man, chimpanzee and crab-eating macaque. The topography of the claustrum is consistent in the three primate species (Figure 1), including its well-established relationships with the adjacent structures (*capsulae*, basal ganglia, insular cortex, thalamus, ventro-lateral temporal cortex). The relative lesser development of the temporal lobe (and especially of the *gyri temporalis medius* and *inferior*) in the chimpanzee and crab-eating monkey places the claustrum of these two species topographically closer to the ventral surface of the brain than in man. The planes of section represented in Figure 1 were regularly applied to the analysis of the chemical neuroanatomy of the claustrum in the three primate species. For cellular studies and practical purposes, in each plane of section the claustrum was furtherly topographically subdivided into dorsal, central and ventral claustrum (for discussion on the possible subdivisions of the claustrum see Hinova-Palova et al., 2013).

The morphological features and the distribution pattern of the PV-ir, CR-ir, NPY-ir and SOM-ir neurons observed in the insular cortex and in the putamen in each section were considered the standard reference control (data not shown). CB immunoreactivity was not detected in our samples while CB-ir neurons were found (data not shown) in archival samples of the rat brain cortex used as positive controls. The relative density of the immunostained neurons is reported in Table 1. The cell density of the PV and CR immunostained neurons in the human, chimpanzee, and crab-eating monkey claustrum, calculated as the total of cells per mm^2^, is reported in Table 2.

**Table 1:** **Distribution and density pattern of CR-, PV-, SOM-, and NPY-ir cells in the dorsal, central, and ventral regions of the claustrum**.

	**Human**			**Chimpanzee**			**Crab-eating macaque**		
	*Dorsal*	*Central*	*Ventral*	*Dorsal*	*Central*	*Ventral*	*Dorsal*	*Central*	*Ventral*
**CR**	+	+++	++	+	+++	++	+	+++	++
**PV**	+	+++	++	+	+++	++	+	+++	++
**SOM**	+	+	+	–	–	–	+	+	+
**NPY**	++	++	++	++	++	++	++	++	++

*+ Moderate; ++ Numerous; +++ Very numerous*.

**Table 2:** **CR and PV neuronal density in the human, chimpanzee, and crab-eating macaque claustrum (count of labeled cells *per* mm^2^)**.

	**CR**			**PV**		
	Cells	mm^2^	Density (cells / mm^2^)	Cells	mm^2^	Density (cells / mm^2^)
**Human**	1070	218	4.9	401	200	2
**Chimpanzee**	380	82.6	4.6	158	78.4	2
**Crab-eating monkey**	134	30.8	4.4	216	32.2	6.7

We observed several morphologically distinct types of immunostained cells in the claustrum of the three species. CR-ir neurons were the most numerous CBP-type expressed, and represented a relatively uniform population. PV-ir neurons resulted more numerous in the crab-eating macaque and belonged to two distinct cell types according to the soma shape and diameter (see below). Immunoreactivity to CR, PV, NPY and SOM was observed in neuronal cell bodies and processes distributed throughout the anterior-posterior extent of the claustrum. Both CR- and PV-ir neurons exhibited a gradient pattern of increasing number from dorsal to ventral claustrum. Differently, NPY and SOM immunostained cells were evenly scattered all through the claustrum (Figure 12).

**Figure 2:**
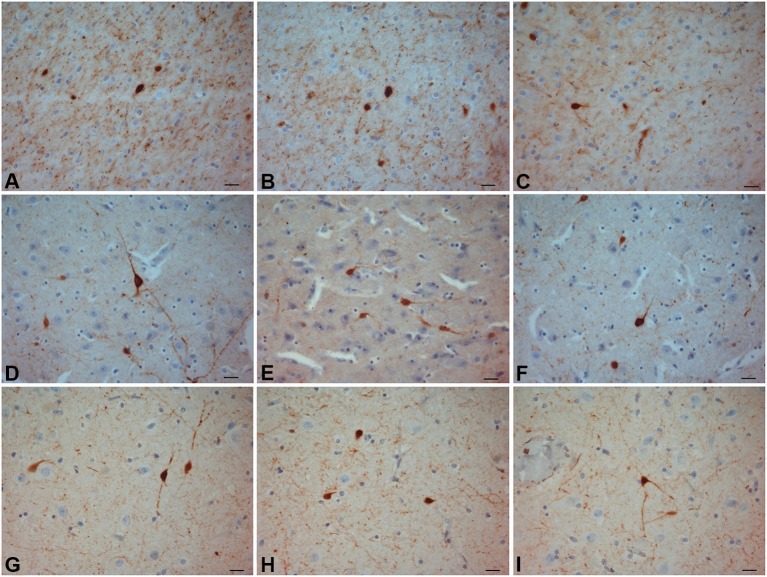
**CR-ir neurons**. **(A–C)** crab-eating monkey; **(D–F)** chimpanzee; **(G–I)** human. Scale bars = 20 µm.

**Figure 3:**
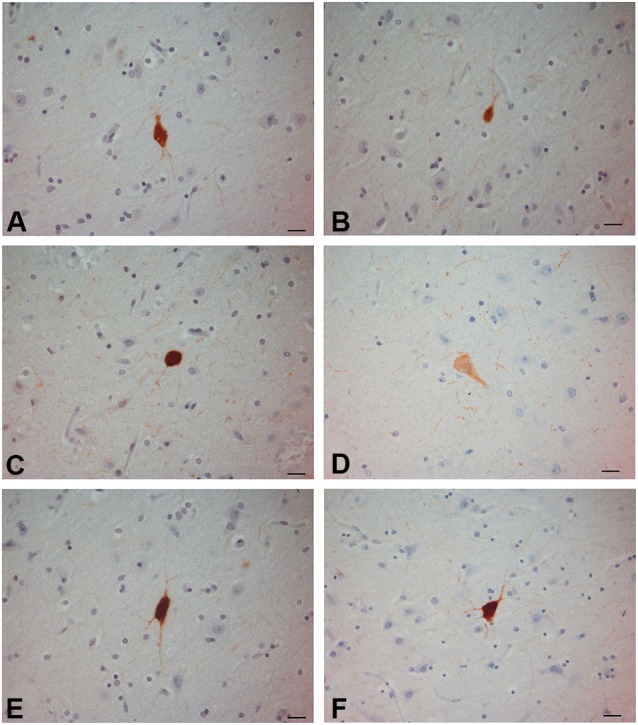
**PV-ir neurons in the human claustrum**. **(A–F)** Scale bars = 20 µm.

**Figure 4:**
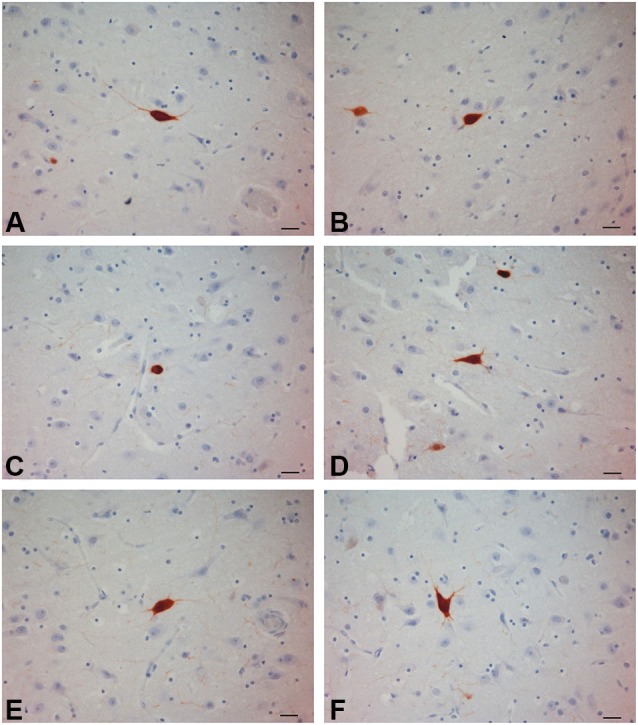
**PV-ir neurons in the chimpanzee claustrum**. **(A–F)** Scale bars = 20 µm.

**Figure 5:**
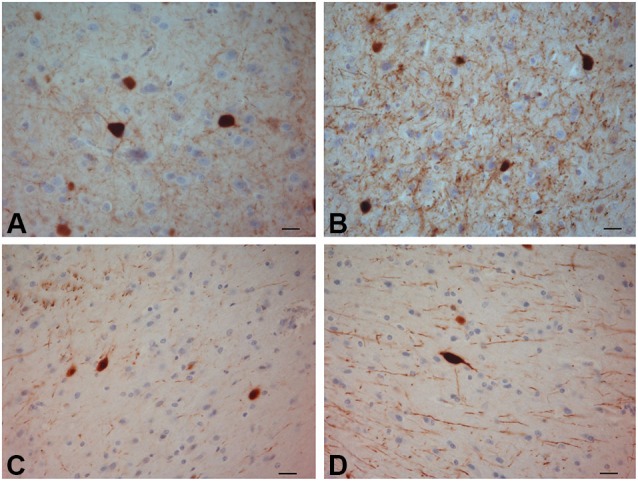
**PV-ir neurons in the crab-eating monkey claustrum**. **(A–D)** Scale bars = 20 µm.

**Figure 6:**
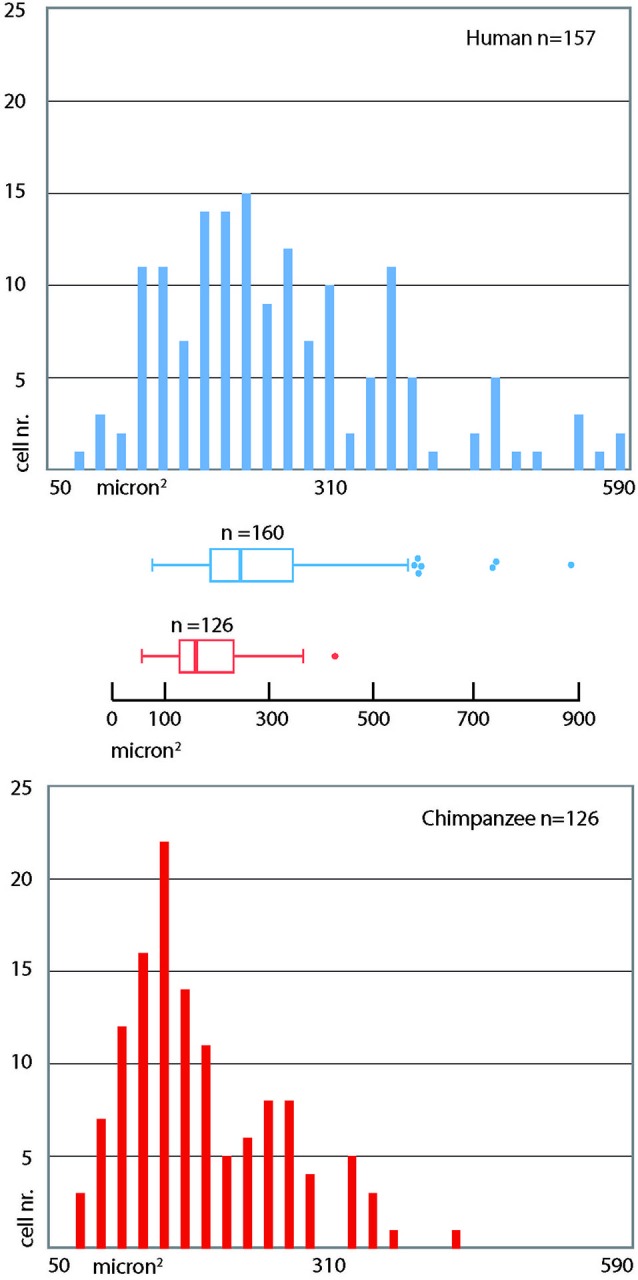
**Frequency distribution of cross-section areas of PV immunoreactive neurons in the human and chimpanzee claustrum**. Bins = 20 µm^2^. The box in the box plot shows the median and interquartile range. Left whiskers include low extreme values; the length of right whiskers is interquartile range × 1.5.

**Figure 7:**
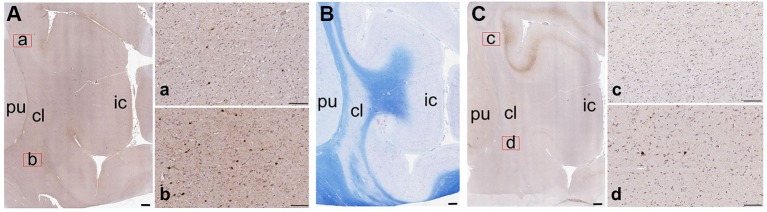
**Low-magnification images of the CBPs in the human claustrum.** **(A)** CR-immunoreactive neurons; a and b are enlargements of the corresponding red rectangles in **A**; **(B)** Reference image stained with Luxol Fast Blue; **(C)** PV-immunoreactive neurons; c and d are enlargements of the corresponding red rectangles in **C**; pu; putamen; cl: claustrum; ic: insular cortex. Scale bars: A, B, C = 1 mm; a, b, c, d, = 100 µm.

**Figure 8:**
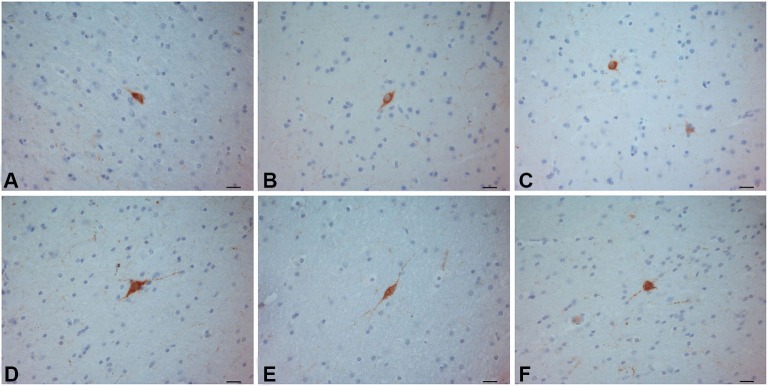
**SOM–ir neurons in the crab-eating monkey (A–C) and human (D–F) claustra.** Scale bars = 20 µm.

**Figure 9:**
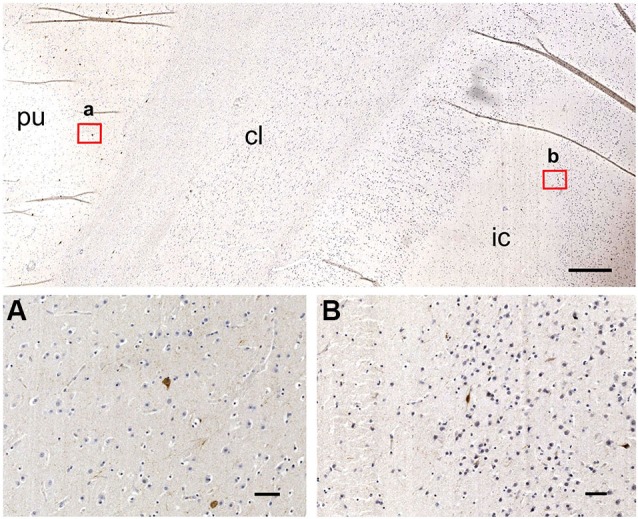
**SOM–ir neurons are absent from the chimpanzee claustrum (top), but present in putamen (A) and insular cortex (B)**. pu; putamen; cl: claustrum; ic: insular cortex. Scale bars; image at the top = 500 µm; a, b = 50 µm.

**Figure 10:**
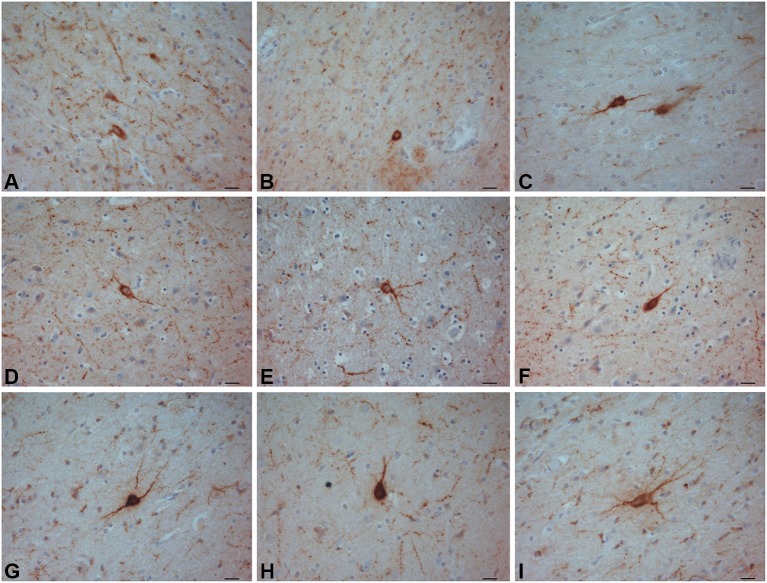
**NPY-ir neurons in the crab-eating monkey (A–C) chimpanzee (D–F) and human (G–I) claustra.** Scale bars = 20 µm.

**Figure 11:**
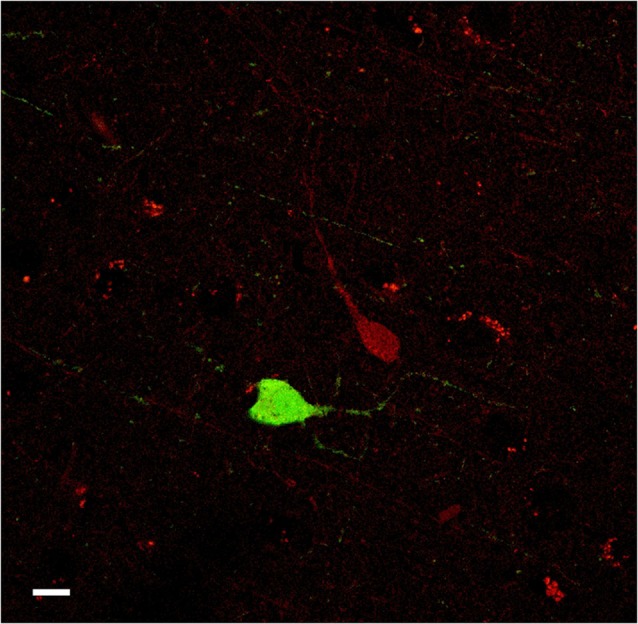
**Confocal microscope images of PV-ir (green), and CR-ir (red) neurons in the human claustrum**. No co-localization was observed. Scale bar = 10 µm.

**Figure 12:**
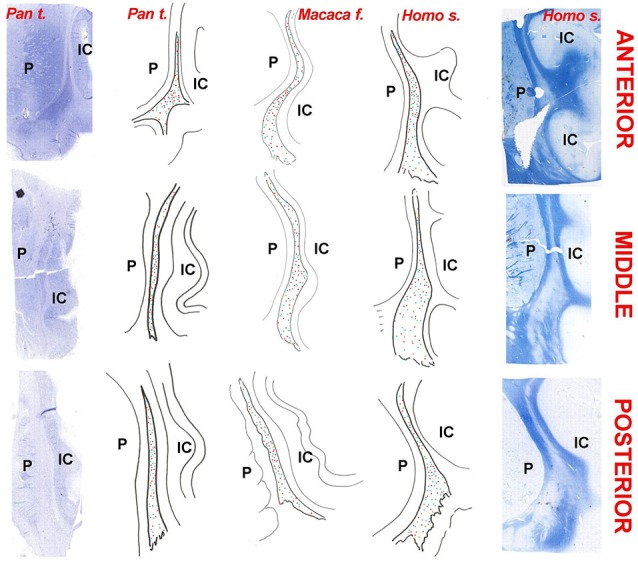
**Chartings of the claustrum of the chimpanzee (*Pan troglodytes*), crab-eating macaque (*Macaca fascicularis*), and man (*Homo sapiens*)**. Column of photographs on the left: Nissl stain of the neurons in the claustrum of the chimpanzee; Column of photographs on the far right: Luxol fast-blue stain of fibers in the human claustrum. The chartings are drawn from original sections. The species is indicated on the top. Red dots: PV; Blue dots: CR; Green dots: NPY; Indigo dots: SOM (absent in the chimpanzee); P = putamen; IC: insular cortex.

### Calretinin

The most frequently observed CR-ir neurons in human, chimpanzee and crab-eating monkey appeared to be darkly stained with 1–2 processes and a round or fusiform somata (Figure 2). The main diameter was inferior to 20 µm. Triangular somata were seldom observed in the human claustrum (Figure 2I). In the three species CR-ir neurons were more abundant in the central and ventral region, few CR-ir neurons were detected in the dorsal part of the claustrum. Positive fibers were localized in the neuropil, in particular they were numerous in the crab-eating monkey (Figures 2A–C).

### Parvalbumin

PV-immunoreactive neurons of different sizes and shape were diffused in the claustrum of the three species (Figures 3–5). In the human and chimpanzee claustrum we described fusiform, round, triangular, polygonal and pear shaped cell bodies (Figures 3, 4). In the crab-eating monkey, somata were mainly fusiform, round or pear shaped (Figure 5). As a rule, labeling in the processes was fainter than in the cell body. In the three species PV-ir neurons were particularly numerous in the central and ventral region. In the dorsal part PV-ir neurons were rarely seen. PV-ir fibers were found in the claustrum neuropil of the crab-eating monkey (Figure 5), but their presence was less conspicuous in the human and chimpanzee. The frequency distribution of cross-section areas of PV labeled neurons in the human and chimpanzee claustrum is shown in Figure 6. For the chimpanzee, there is a main population of cells with mean area around 190 µm^2^, and a second population with a peak of frequency around 300 µm^2^. The distribution of cross-section areas is more widespread in humans, compared to the chimpanzee.

Figure 7 contains low-magnification comparisons of the distribution of CR- (Figure 7Aa-b) and PV- (Figure 7Cc-d) immunoreactive neurons in the human claustrum.

### Somatostatin

In the human and crab-eating monkey claustrum, SOM positive somata were identified (Figure 8). They presented a round or fusiform soma with faintly labeled processes. SOM immunoreactive fibers were rarely seen. These immunostained neurons were evenly scattered throughout the human and monkey claustrum. In the chimpanzee claustrum, SOM-ir neurons were not observed (Figure 9), but neurons clearly immunostained were detected in the putamen (Figure 9A) and in the insular cortex (Figure 9B).

### NPY

NPY-ir neurons were found in the claustrum of the three species (Figure 10). Neurons with darkly stained somata and processes were uniformly diffused throughout the claustrum. The soma was round, fusiform or triangular. Many positive beaded fibers were localized in the neuropil.

### Co-localization

The number of cells examined was approx. 8–12 for each slide (total 16–20 cells for each species), depending on the condition of the single tissue sections. No co-localization of PV with CR was found. PV- and CR-ir cells appeared to be organized into two different neuronal populations (Figure 11).

## Discussion

The present study describes the localization and the morphology of the PV-, CR-, NPY-, and SOM-ir neurons in the human, chimpanzee and crab-eating monkey claustrum. Data on the presence of selected CBPs in the human and monkey claustrum were already present in the literature (for general reference see the elegant study of Hinova-Palova et al., 2013 on PV in the human claustrum). However the present study describes for the first time the CBPs in three key primates. Furthermore, to our knowledge, this is the first time that the localization of CR, PV, SOM, and NPY has been studied in chimpanzee claustrum.

In general, the topography of the claustrum, the morphological feature and distribution of the labeled cells were similar in the three species, confirming their close phylogenetic relationship. Here we assume that the human and chimpanzee dorsal claustrum maintains the extensive relationship with the somatosensory and with the auditory cortical areas demonstrated in the Rhesus monkey (Minciacchi et al., 1991; Remedios et al., 2010). Moreover, the segregation of the visual modality in the ventrocaudal claustrum, as described in the macaque monkey (e.g., Remedios et al., 2010), is likely to be preserved also in the enlarged ventral part of the ape and human claustrum. It remains to be established whether the higher density of PV-ir neurons in the ventral claustrum, observed in the present study, is functionally related to the processing of visual (rather than somatosensory and auditory) information.

In our immunohistochemical characterization, as seen in coronal sections, ir-neural cells were always denser in the central part of the dorso-ventral extension of the claustrum, but we cannot presently pinpoint any specific function to these data, as classical projection studies obviously do not apply to apes and human. Interestingly, a novel approach to mapping claustral projections in living humans, using constrained spherical deconvolution tractography (Milardi et al., 2013), showed multiple and possibly multi-functional connections of the claustrum with cortical and subcortical structures. On the contrary, studies performed in rats by proteomic analysis combined with traditional tracing methods (Mathur et al., 2009) showed that claustral connections were limited to cortical structures, and no sub-cortical projection was identified.

The major difference that we found among the three species was the absence of SOM-ir neurons in the claustrum of the chimpanzee. Technical or fixation-related motives for the absence of SOM staining in the chimpanzee may be excluded, since SOM-ir neurons were clearly present in the adjacent structures: the putamen and the insular cortex. It is possible that this difference could be due to species-specific variability. On the other hand, neurochemical variations among closely related species are not uncommon, i.e., the expression of cortical chemical markers varies among rodents (Xu et al., 2006; Gonchar et al., 2008; Miyoshi et al., 2007).

On the whole, our data agree with those reported for the presence of PV-ir and CR-ir neurons in the claustrum of different species (Reynhout and Baizer, 1999; Real et al., 2003; Wojcik et al., 2004; Rahman and Baizer, 2007) including man (Hinova-Palova et al., 2013). In particular, our findings on the PV-ir neurons in the human claustrum were consistent with those previously reported by Hinova-Palova et al. (2013). The technique we employed (paraffin sections of 5 µm) did not allow us to describe the dendritic arborization but the PV-ir cell distribution agrees with what observed by these authors. We were not able to distinguish the existence of seven morphological subtypes of PV-ir neurons, but we identified the presence at least of five subtypes in the human claustrum: round, fusiform, triangular, polygonal and pear shaped. The morphology of the cells expressing CR was similar to that reported in the monkey (*Macaca fascicularis*) (Reynhout and Baizer, 1999). Both the PV- and CR-labeled were more abundant in the central and ventral part of the claustrum, as reported in Table 1.

Different neuronal types have been described in the human claustrum by Golgi stain (Braak and Braak, 1982). According to the soma size and shape, we can speculate that the PV-ir cells of our study may correspond—at least in part–to the large aspiny neurons described by Braak and Braak (1982) with Golgi stain. The CR-ir described in the present paper may match small aspiny cells of Golgi stain, even though our observations were not consistent with the dendrites radiating in all directions as reported in the cited study (Braak and Braak, 1982). The CR-ir cells may also represent interneurons not yet described by Golgi technique.

We note that CB immunoreactivity was not detected in our samples, but the rat brain cortex used as positive control (data not shown) tested positive. In addition, the CB monoclonal antibody used in the present study has been shown to immunoreact with primate retinal cells (Puthussery et al., 2011). Possible explanations for the lack of CB immunoreactivity in our samples include potential loss of signal due to post-mortem interval occurred before sampling or an hitherto undetermined specific factor influencing the presence of CB in the primate claustrum. CB is widely expressed by several classes of cortical interneurons, such as neurogliaform, double bouquet, and Martinotti cells (e.g., Gabbott and Bacon, 1996). The lack of CB-ir neurons can be explained by the absence of some claustral interneuron populations homologue to those of the cortex. However, we cannot rule out that the absence of CB can be due to the fact that its peculiar role in Ca^2+^ buffering and signaling (extensively discussed in Bastianelli, 2003) is not needed in the primate claustrum.

Calcium-binding proteins and neuropeptides have been used as markers that distinguish among cortical interneurons (Garcia-Segura et al., 1984; DeFelipe et al., 1989; Hendry et al., 1989; DeFelipe, 1993; Cauli et al., 1997; Gonchar and Burkhalter, 1997; Somogyi and Klausberger, 2005; Ascoli et al., 2008). Moreover, it has been demonstrated that inhibitory neurons expressing CBPs contain also neuropeptides such as: SOM, VIP, CCK, and NPY (Xu et al., 2006). In particular, PV, CR and SOM are considered informative markers because they have minimal overlap with other markers (DeFelipe, 1993; Kubota et al., 1994; Kawaguchi and Kubota, 1996, 1997; Gonchar and Burkhalter, 1997; Gonchar et al., 2008). In the present study we identified SOM and NPY labeled neurons in the investigated claustra with the exception of SOM in the chimpanzee. Former studies confirmed the presence of these two neuropeptides in the rat claustrum (Kowianski et al., 2001, 2009). The somewhat puzzling absence of SOM-ir elements in the claustrum of the chimpanzee (Figure 9) remains unexplained, considering also that SOM-ir neurons are present in the putamen and insular cortex (Figures 9A–B). We were not able to trace any former study on the expression of SOM in the chimpanzee brain. A study in the human (Mengod et al., 1992), failed to detect SOM mRNA-containing neurons in the claustrum, but described them in several locations, including the neocortex and putamen. Another study (Breder et al., 1992) reported the presence of SSTR2 (SOM receptor type 2) mRNA but not SSTR1 (SOM receptor type 1) mRNA in rats and humans, suggesting the existence of a complex network (for review see Møller et al., 2003). A study of the expression of the different SOM receptors in the chimpanzee may contribute to a better understanding of our data.

The presence of CBPs, SOM and NPY, along with the lack of PV-CR co-localization, suggests the existence of diverse neuronal subpopulations in the chimpanzee, crab-eating monkey and human claustrum. This scheme could indicate a similarity to the neocortex where different non-overlapping classes of GABAergic interneurons have been described (DeFelipe et al., 1989; Hendry et al., 1989; DeFelipe, 1993; Cauli et al., 1997; Gonchar and Burkhalter, 1997; Kawaguchi and Kubota, 1997; Somogyi and Klausberger, 2005; Ascoli et al., 2008). Similarly, several classes of claustral inhibitory interneurons can be distinguished on the basis of the differential expression of CBPs and neuropeptides (see also Kowianski et al., 2009; Smythies et al., 2012). However, we should also take into account that CBPs are not exclusively expressed by GABAergic interneurons of the claustrum, but are also found in spiny projection neurons (Hinova-Palova et al., 2007; Hendry et al., 1989).

Is the neurochemistry of the primate claustrum different form that of other mammals? Among the three species we noted a difference in the density of the neurons expressing the CaBPs (Table 2). In particular, in the human and in the chimpanzee the density of the CR-ir neurons was greater than that of the cells expressing PV while in the macaque there was an opposite situation. Several studies have been carried out in non-primate mammals. Real et al. (2003) investigated the CBPs expression in the dorsal and ventral (endopiriform nucleus, ED) division of the mouse claustrum. CR-ir neurons were very scarce in both divisions. In contrast, PV-ir cells were more numerous in the dorsal division than in the ED. These results were in line with the findings reported in the rat claustrum (Celio, 1990; Druga et al., 1993). Four neuronal types have been described in the rabbit claustrum based on CBPs immunohistochemistry (Wojcik et al., 2004). In this latter species, PV immunoreactivity in the ED was lower than that observed in the dorsal claustrum while immunostain for CR was low in both divisions, similarly to what reported for the mouse (Real et al., 2003). As we reported in the crab-eating monkey, in the cat, cells immunoreactive to CR were less numerous than those positive to PV (Rahman and Baizer, 2007). Our results in the human and chimpanzee suggest the opposite, since, CR-ir neurons represented the most evident CBP-positive category. Furthermore, both PV- and CR-ir cells were more concentrated in the central and ventral part of the claustrum. Most likely, these differences were due to species-specific differences (Baimbridge et al., 1992), and possibly indicate a neurochemical organization peculiar of primates.

A comparison of the morphology and the cellular density observed in the human claustrum with that of the mammalian neocortex may yield important information and contribute to the understanding of the ontogenesis of this enigmatic structure. In the mammal neocortex, CR-ir neurons were mainly bipolar or bitufted, displayed a fusiform or oval soma, and localized in layers II and III (Barinka and Druga, 2010). Differently, the greatest density of PV-ir positive neurons of the first type with large round multipolar somata and of the second type with a small- to medium-sized multipolar soma has been described in layers III and IV. Besides, in the mammalian (and especially in the primate) neocortex the relative density of CR-ir cells was approximately twice that of PV-ir elements (Hof et al., 1999). In particular, the CR/PV ratio in the monkey was found to be about 1.8 (Barinka and Druga, 2010). These findings are in agreement with our results described in Table 2. As the former Authors reported in the neocortex, we described a single class of CR-ir cells and, at least in the chimpanzee, two types of PV-ir neurons, based on the size. Unlike the cortex, these cells were evenly distributed without a layered organization. Furthermore, we observed a higher density of the CR-ir in respect to PV-ir cells. The latter data is close to that indicated in the neocortex. Taken together these findings may give a further contribute to the pallial origin (and possibly function) of the human claustrum (for a comprehensive review see Park et al., 2012; Pirone et al., 2012). At least in the cortex, there is a considerable degree of CR-VIP overlap (e.g., Cauli et al., 1997) and VIP interneurons mediate the feedback input from higher-order cortical areas (reviewed in Karnani et al., 2014). Therefore, the prevalence of CR neurons in the primate cortex and claustrum is likely to be justified by the substantial increase of the associative network in the primate brain.

Based on neuronal morphology and present failure of co-localization, our study suggests that in the primate claustrum CR- and PV-containing neurons might be segregated into two distinct sub-populations. These findings agree with what previously reported for human and monkey cortical interneurons (del Rio and DeFelipe, 1996; Zaitsev et al., 2009, 2005). The importance of CBPs as a tool for interneuronal subpopulations sorting has been recently validated by gene cluster analysis (Toledo-Rodriguez et al., 2004). In the neocortex of rodents, 100% of CR-labeled neurons were GABAergic (Gonchar and Burkhalter, 1997; Gonchar et al., 2008). This does not fully apply to primates, in which approx 25% of CR-ir neurons are not GABAergic (del Rio and DeFelipe, 1996; Melchitzky et al., 2005). Moreover, PV is reported to be consistently localized in subpopulations of GABAergic neurons in the central nervous system (Bastianelli, 2003). Based on the studies mentioned above, we may suppose that in the human claustrum a large part of PV-ir and CR-ir neurons belong to the GABAergic system too. The presence of two different interneuronal sub-populations could indicate the existence of an inhibitory and a disinhibitory network able to modulate complex intra or extra-claustral interactions. This is consistent with the theory of the claustrum function proposed by Crick and Koch (2005) and with the findings reported by Rahman and Baizer (2007) in the cat. Although studies on the human and macaque claustrum were already present in the literature, the present investigation reports the first description of the claustrum of an ape, the chimpanzee. We also present evidence of PV- and CR-ir neurons in the chimpanzee claustrum, confirm the distribution of the CBPs in the human and offer for the first time direct comparisons in the three primates. Our findings suggest that these two CBPs are localized into two different sub-populations of PV- and CR-ir interneurons. The morphology and the density of distribution of the immunostained cells further support a possible common ontogeny of the claustrum and the neocortex (Pirone et al., 2012).

## Conflict of interest statement

The authors declare that the research was conducted in the absence of any commercial or financial relationships that could be construed as a potential conflict of interest.

## Footnotes

^1^“Member States should, where appropriate, facilitate the establishment of programmes for sharing the organs and tissue of animals that are killed”.

^2^www.ebi.ac.uk

^3^www.ensembl.org
